# Ffuzz: Towards full system high coverage fuzz testing on binary executables

**DOI:** 10.1371/journal.pone.0196733

**Published:** 2018-05-23

**Authors:** Bin Zhang, Jiaxi Ye, Xing Bi, Chao Feng, Chaojing Tang

**Affiliations:** School of Electronic Science, National University of Defense Technology, Changsha, Hunan, P.R.C.; Beijing University of Posts and Telecommunications, CHINA

## Abstract

Bugs and vulnerabilities in binary executables threaten cyber security. Current discovery methods, like fuzz testing, symbolic execution and manual analysis, both have advantages and disadvantages when exercising the deeper code area in binary executables to find more bugs. In this paper, we designed and implemented a hybrid automatic bug finding tool—Ffuzz—on top of fuzz testing and selective symbolic execution. It targets full system software stack testing including both the user space and kernel space. Combining these two mainstream techniques enables us to achieve higher coverage and avoid getting stuck both in fuzz testing and symbolic execution. We also proposed two key optimizations to improve the efficiency of full system testing. We evaluated the efficiency and effectiveness of our method on real-world binary software and 844 memory corruption vulnerable programs in the Juliet test suite. The results show that Ffuzz can discover software bugs in the full system software stack effectively and efficiently.

## Introduction

Although software quality continues to improve, program errors expose cybersecurity to critical threats. Even though a large proportion of errors is discovered by hand, manual analysis is not scalable for large and complex modern software. Researchers have put a great deal of effort into devising automatic testing methods for large-scale binary software; in the last several years, many automatic methods have been proposed, such as fuzz testing [1] and symbolic execution [2].

Fuzz testing and symbolic execution are mainstream techniques that are used to find software bugs. Fuzz testing expects to discover software exceptions by feeding the target software with randomly mutated test cases. It is the most popular technique since it is easy to deploy. Symbolic execution interprets programs by assigning symbolic rather than concrete data to program inputs [2]. By solving corresponding path conditions, it can generate inputs that can cover specific code regions. Unfortunately, symbolic execution often results in an exponential number of paths that bottleneck the constraint solver.

Fuzz testing still faces problems when testing full system software stack to find low-level vulnerabilities (i.e., bugs in device drivers and OS kernels). For example, AFL [3] is the most popular fuzzing tool and has found many exploitable bugs. However, as mentioned previously, it works only in the user space and cannot deal with the kernel space. DDT [4] is a famous device drive testing tool based on the selective symbolic execution platform S2E [5, 6], but it cannot solve the “path explosion” problem, which results from symbolic loop unrolling.

After analyzing the advantages and disadvantages of fuzz testing and symbolic execution when testing a full system software stack, we realized that symbolic execution has the powerful ability to find “corner” bugs while spending a great deal of time on program analysis and constraint solving. On the contrary, fuzz testing is efficient at testing because it concentrates on random mutations; however, it is inefficient at finding “corner” bugs.

In this paper, we leverage fuzz testing and selective symbolic execution [5] and propose a full system and high-coverage fuzzing tool Ffuzz. By combining the two mainstream techniques, we integrate the advantages and mitigate the disadvantages to find deeper bugs. Ffuzz is not the first tool to combine different testing methods. However, as far as we know, it is the first tool that can perform a coverage-based hybrid test on a full system software stack. Furthermore, we performed an additional evaluation to demonstrate the performance of Ffuzz.

This paper makes the following contributions.

- We propose a new method to perform fuzz testing on a full system software stack based on fuzz testing and selective symbolic execution.
- We leverage two key optimizations to improve the efficiency of full system software fuzz testing.
- We designed and implemented a tool—Ffuzz—and we evaluated Ffuzz on real-world binary software to demonstrate the effectiveness and efficiency from several different viewpoints.

The rest of this paper is organized as follows. Then in Section 2, we review some of the previous work in software testing. Section 3 describes the basic design of Ffuzz. Section 4 addresses the efficient problem of full system fuzz testing and discusses our optimizations. Section 5 reports the implementation of Ffuzz. Section 6 presents experimental evaluation results of the key optimizations and demonstrates the effectiveness and efficiency. Section 7 discusses our contribution and other related work. And Section 8 concludes this paper.

## Related work

In this section, we introduce the research progress of fuzz testing, symbolic execution, and hybrid testing.

### Fuzz testing

Fuzz testing randomly mutates/modifies selected program inputs using several mutation operators (e.g., bit-flip, boundary value substitution, block deletion and duplication) to generate new test cases before feeding them to the target program. Then the target program is executed and monitored to capture abnormal behaviors such as program crashes and abortions. However, since modern software’s inputs are well-structured, a large portion of the randomly generated test cases is rejected by the program, which brings low efficiency problem of traditional fuzz testing.

To address this problem, model-based fuzz testing technique was proposed. And several fuzzing frameworks are released, such as Peach [7] and Spike [8]. This technique leverages the knowledge of input’s structure to *construct* new test cases that have lower probability to be rejected by the program. However, without the knowledge of program internal information, model-based fuzz testing still cannot penetrate into deeper code areas to find more bugs.

Coverage based fuzz testing collects control flow information of the program and leverages such information to guide random testing. For example, AFL captures basic block transitions and coarse branch-taken hit counts into a bitmap by lightweight instrumentation. Then it leverages a *Genetic Algorithm* like mechanism to guide mutations. A test case that triggers new program behaviors (e.g., covers new block transitions) is collected into a seed queue which is used as seed file to mutate. Other test cases are discarded to reduce useless mutations. AFL has exposed many bugs in different softwares. However, since coverage based fuzz testing is still built on top of random mutations, it cannot overcome branches that guarded with complex constraints.

### Symbolic execution

Symbolic execution is an analysis method to determine what input can drive execution to specific code regions [2]. It interprets the program by assigning *symbolic data* rather than actual (*concrete*) data to the program inputs.

Consider a program *P* which consists of a set of program variables *Var* and a set of instructions *Inst*. An ***execution path*** is a serial of instructions, such as *I*_0_ → *I*_1_ → ⋯ → *I*_*n*_, where *I*_*i*_ ∈ *Inst*. Unlike execution path, which focuses on instructions, a ***program state*** concentrates the value of each variable. Since the bits of register and memory are bounded, the number of program state can be enumerated. However, the total number of execution path may be infinite, which is also known as “path explosion” problem.

Dynamic symbolic execution maintains a ***symbolic state*** *s* = 〈*I*, *M*, *S*, *pc*〉, where *I* ∈ *Inst* denotes the next instruction to be executed, *M* is the concrete memory store which maps *Var* to concrete data, *S* is the symbolic memory store which maps *Var* to symbolic expressions, and *pc* is the symbolic path constraint which is a first order quantifier free formula over symbolic expressions. Since each symbolic state can be individually mapped to an execution path, we can use *symbolic state* to equivalently represent an execution path.

To ease the “path explosion” problem, selective symbolic execution [5] takes a different approach, by interleaving portions of code that are concretely run with fully symbolic phases. Such an approach enables the symbolic execution only focuses on interesting code regions. This can reduce the total number of symbolic states. However, since symbolic execution is a path-sensitive analysis method, selective symbolic execution still faces “path explosion” problem when testing some code structures such as symbolic loops and symbolic pointers.

### Hybrid testing

Full system testing is challenging because most testing tools cannot gain full access to low levels of the software stack. To deal with this, virtualization technology is often utilized in full system testing tools. QEMU [9] is a typical virtualization tool that is widely used in current full system testing tools such as Bitblaze [10], S2E [6], vUSBf [11], DDT [4], and SymDrive [12]. However, full system testing, especially within the virtual machine, is more expensive than testing only in the user space. In other words, full system fuzzing has to solve the low-efficiency problem. vUSBf tries to improve efficiency by utilizing multiple QEMUs to speed up the test procedure. In our design of Ffuzz, we have proposed three key optimizations to make Ffuzz more efficient.

As mentioned previously, Ffuzz is not the first tool to combine fuzz testing and symbolic execution. Hybrid fuzz testinging [13] uses symbolic execution to discover frontier nodes that represent unique paths of the program. After collecting as many frontier nodes as possible under a user-specifiable resource constraint, it transitions to random inputs. This tool focuses on binaries but only performs a one-time transition between symbolic execution and fuzz testing. Hybrid Concolic Testing [14] implements multiple transitions between symbolic execution and fuzz testing. However, because it is built on top of CUTE [15], a source code oriented testing tool, hybrid concolic testing still cannot be deployed on binary testing directly. Driller [16] is an up-to-date hybrid testing tool that leverages fuzz testing and selective concolic execution in a complementary manner to find deeper bugs. It is more practical when compared with previous hybrid tools. It supports detection for any vulnerabilities that leads to a program crash, whereas other previous techniques supports very specific types of vulnerabilities. Meanwhile, existing hybrid techniques do not take full advantages of both fuzz testing and concolic execution, and they are also affected by the “path explosion” problem. Driller alleviates this by limiting the total number of states forked from concolic execution engine. However, Driller only focuses on programs in the user space; it still cannot be leveraged for full system software stack testing. MAYHEM [17], the champion of the DARPA Cyber Grand Challenge [18], shows the great potential of hybrid testing methods. However, MAYHEM is not a whole-system symbolic executor, it has not made models for all system/library calls and cannot analyze kernel/library code that is not modeled. Other tools try to make full use of symbolic execution to maximize the code coverage; they collect symbolic constraints placed on each input and then negate these constraints to generate a new test case that will take another uncovered path, such as SAGE [19], Dowser [20], and FuzzWin [21]. However, these tools execute each input in symbolic mode, which determines that they have to face the path explosion problem. And, worse, some constraints are time consuming for state-of-the-art constraint solvers, like solving the checksum.

## Basic architecture of FFuzz

**Fig 1:**
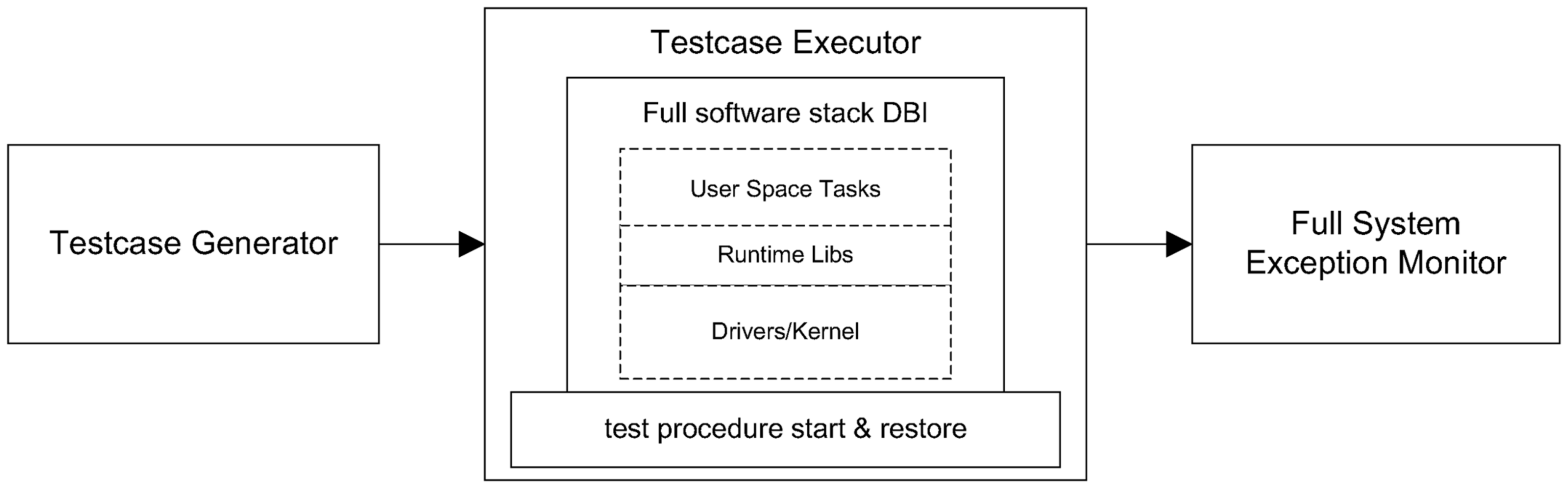
Framework of Ffuzz at a high level scope.

From a high level perspective, Ffuzz is a full system guided fuzz testing platform, and it consists of three basic components, i.e., test case generator, test case executor, and full system exception monitor, as shown in Fig 1.

The test case generator is built on top of the off-the-shell genetic mutator. In the following part of this section, we will focus on the details of the test case executor, i.e., the full software stack DBI and test procedure start & restore and the full system exception monitor.

### Full software stack DBI

Different test cases may map to the same program path, which bottlenecks traditional fuzz testing. To ease this problem, we leverage the genetic-like mutation of AFL. AFL takes advantage of the TCG module [22] in QEMU [9] to record the covered paths. However, it misses the paths of the Ring 0 layer. We utilize full system mode QEMU to solve the full software stack recording problem. As QEMU simulates the execution of the whole system, we first extract the OS-level semantics, such as processes, threads, and interruptions. After obtaining the current task information, we can limit the instrumentation only to target the task and ignore the others.

### Fast test procedure restore

When working in the user space, the testing procedure always starts by relaunching the program. However, in full system mode, the testing procedure has to restore the full system to keep the testing environment consistent for each test case. Obviously, restoring the whole system from the very beginning is too expensive. In the design of Ffuzz, we leverage the superiority of symbolic execution to solve this problem.

**Fig 2:**
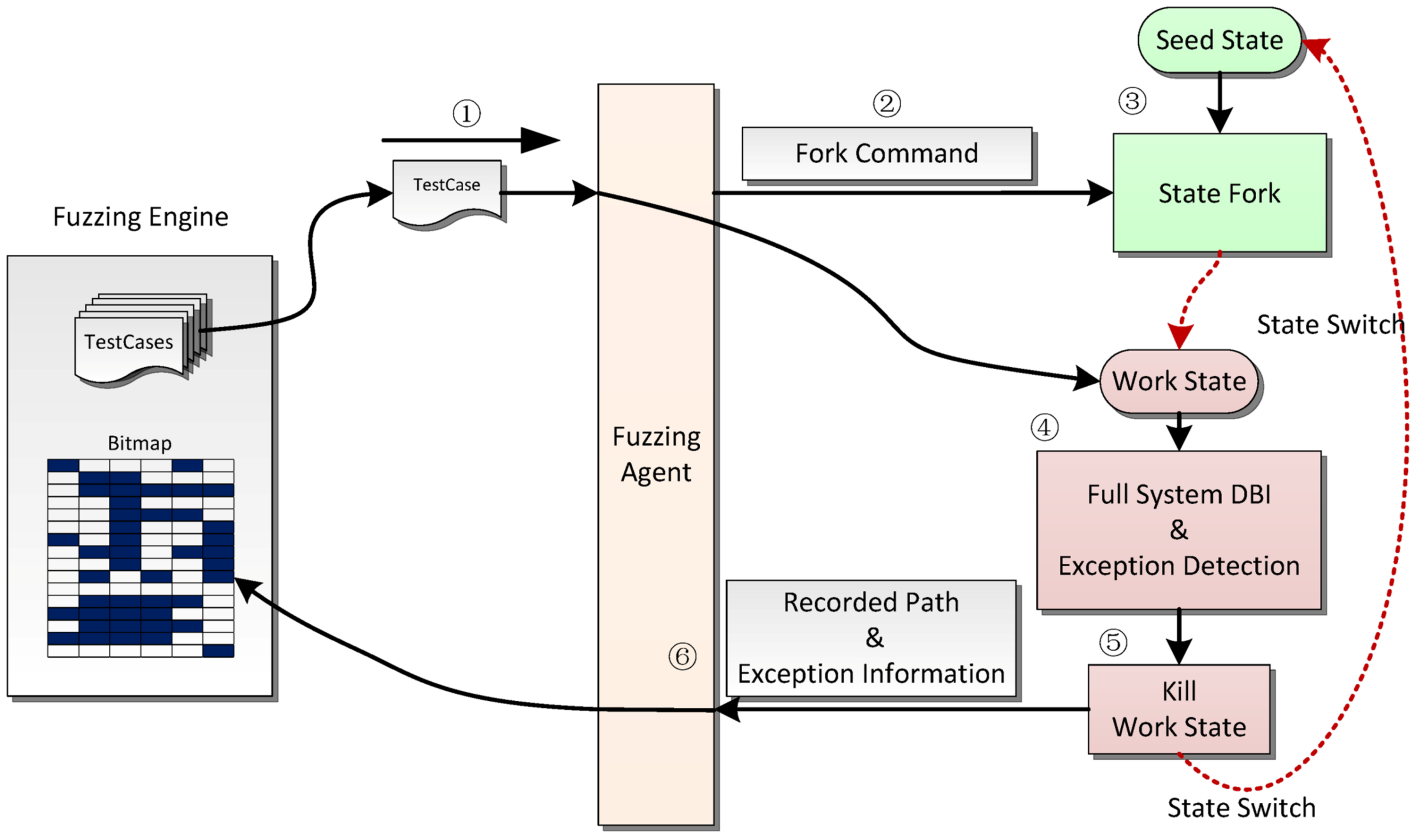
Fuzzing agent framework.

We take the advantage of fast state cloning and transition in symbolic execution to start and restore a test procedure quickly. To achieve this, we design a fuzzing agent in the guest OS of QEMU to interact with the fuzzing engine. The framework of the fuzzing agent is shown in Fig 2; the fuzzing agent is used to transfer new test cases as well as the corresponding events from the fuzzing engine to QEMU and return the recorded path and exception information.

Once a new test case is generated (marked as 1), the fuzzing agent copies it to the guest OS and also emits a fork command (marked as 2) to instruct QEMU to fork and switch to a new work state (marked as 3). The state forking in the symbolic execution engine is a *light-weight* *copy-on-write* operation, which reduces the overhead of restarting the full system from the very beginning of a snapshot. After switching to the work state, the full system DBI and exception detector in QEMU starts to record executed paths and captures abnormal behaviors. Once the target task has been detected as having exited or the testing time exceeds a given time budget, the work state is terminated, and the execution flow switches back to the seed state to wait for the fork command again (marked as 5). Meanwhile, the recorded path is collected to update the fuzzing engine’s bitmap, and the exception information is transferred for future analysis (marked as 6).

### Full system exception monitor

Exception detection is a core component of the fuzz testing framework. There are many ways to track program exceptions, such as attaching a debugger or using ptrace in a Unix system to track the execution of the target program. Using traditional exception detection methods may create overhead when applied to the full system emulation approaches like QEMU (it is necessary to execute the extra code of the debugger or the parent code used for ptrace).

In the *nix system, once CPU generates an exception, such as a Divide Error, the kernel sends the process that raises the exception a signal to notify it of an abnormal condition (i.e., SIGFPE is sent in case of a Divide Error). After receiving this exception signal, the abnormal process takes measures to handle this signal or just takes the default handle.

For user space crashes, we intercept the process exit function of the kernel (i.e., do_exit). A do_exit invocation means a task is about to be terminated, and the invocation argument donates the terminate code. We can derive the corresponding exception type as mentioned above by analyzing the terminate code. For kernel space crashes, we intercept the entrance function of the *kernel panic* handler (i.e., oops_enter), which indicates that serious errors had occurred in the kernel. The addresses of both *do_exit* and *oops_enter* can be obtained from the *System.map* file directly.

Once an abnormal behavior is detected, the current work state is terminated, and the exception information is transferred to the fuzzing engine for future analysis as well as the abnormal test case.

## Key optimizations

Although we have leveraged the Fast Test Procedure Start & Restore to ease the overhead of full system emulation, Ffuzz still faces the low efficiency problem since testing in a full system has to execute irrelevant environment code. To deal with this problem, we introduce two key optimizations into Ffuzz.

### Fast path discovery

Reducing the number of execution times can reduce overhead. Based on this, we have proposed a novel approach—Fast Path Discovery(*FPD*) to discard **redundant** test cases. A test case is redundant when it steers the program executing a path that has been covered previously. Redundant test cases gain no coverage improvement but bring overhead.

The kernel insight of our approach is that whether a test case is redundant can be predicated by path constraint verification without being executed.

Our approach is built on top of a basic hypothesis.

Given a deterministic program *P* and two inputs *A* and *B* (*A* and *B* have the same size). Let *P*_*A*_ and *P*_*B*_ denote the execution path of *A* and *B* in *P*, respectively; and *PC*_*A*_ refers to the path constraint of *P*_*A*_. If *B* satisfies *PC*_*A*_ (i.e., *PC*_*A*_(*B*) = *TRUE*), then *B* will steer *P* to execute the same path with *A* (i.e., *P*_*A*_ = *P*_*B*_). If program *P* is *deterministic*, the path remains the same when testing with the same inputs for arbitrary times.

The motivated code in Listing 1 demonstrates our approach.

**Listing 1.** A motivating code for fast path discovery.

**int** main (**int** argc, char* argv){

 FILE * infile;

 **if** ((infile = fopen (argv [1], READ_BINARY)) == NULL)

  exit (EXIT_FAILURE);

 **int** c1, c2;

 c1 = read_1_byte (infile);

 **if** (c1 != 0xFF)

  assert (0 && “Bad Start”);

 c2 = read_next_byte (infile);

 **if** (c2 != 0xD8)

  assert (0 && “Not a target file”);

 **unsigned int** length;

 length = read_next_2_bytes (infile);

 **if** (length < 2)

  ERREXIT(“Erroneous file marker length”);

 length −= 2;

 printf (“Target file length is %d. \n”, length);

 fclose (infile)

 **return** 0;

}

**Fig 3:**
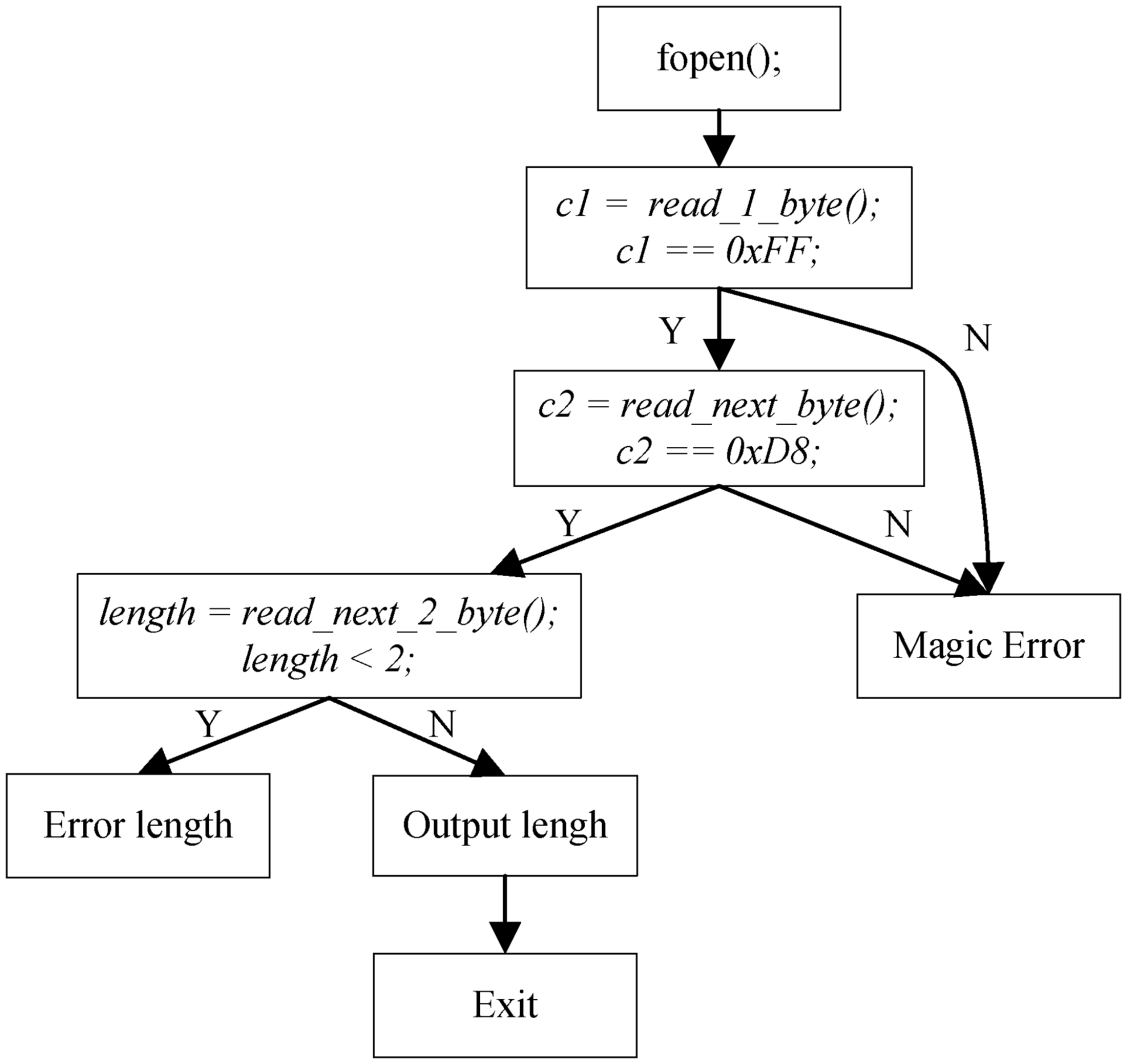
CFG of motivated code.

This sample code snippet is extracted from a real-world JPEG parsing program. It first tries to open the file specified by the argument with binary read-only mode. If the file is opened successfully, the program then checks the magic number of this file (∖xFF∖xD8 represents a JPEG file). After successfully checking the magic number, the length of this file is obtained by invoking read_next_2_bytes, and then the real length is calculated and printed. Fig 3 shows the control flow graph of this motivated program.

Normally, the fuzzing engine starts with a random input test case. Assume that the initial input is ∖xAA∖xBB∖xCC∖xDD, Table 1 lists the process of our approach.

**Table 1:** Sample procedure of *FPD*.

Step	Test Case	Covered Paths	Redundancy
1	∖xAA∖xBB∖xCC∖xDD	{*off*_0_! = 0xFF}	F
2	∖xAC∖xCD∖xC1∖x57	{*off*_0_! = 0xFF}	T
3	∖xFF∖xB3∖x7A∖xE3	{*off*_0_! = 0xFF} {*off*_0_! = 0xFF ∩ *off*_1_ == 0xD8}	F
4	∖x90∖xB3∖xB6∖xF3	{*off*_0_! = 0xFF} {*off*_0_! = 0xFF ∩ *off*_1_ == 0xD8}	T
5	∖xFF∖xD8∖xB1∖xFF	{*off*_0_! = 0xFF} {*off*_0_! = 0xFF ∩ *off*_1_ == 0xD8} {*off*_0_! = 0xFF ∩ *off*_1_ == 0xD8 ∩ *off*_2&3_ >= 2 }	F
6	∖xFF∖xD8∖x5A∖x1F	{*off*_0_! = 0xFF} {*off*_0_! = 0xFF ∩ *off*_1_ == 0xD8} {*off*_0_! = 0xFF ∩ *off*_1_ == 0xD8 ∩ *off*_2&3_ >= 2 }	T
7	∖xFF∖xD8∖x00∖x00	{*off*_0_! = 0xFF} {*off*_0_! = 0xFF ∩ *off*_1_ == 0xD8} {*off*_0_! = 0xFF ∩ *off*_1_ == 0xD8 ∩ *off*_2&3_ >= 2 } {*off*_0_! = 0xFF ∩ *off*_1_ == 0xD8 ∩ *off*_2&3_ < 2 }	F

Each input test case is initially treated as significant; after executing the sample code 1 with the first input, the covered path is updated to ((*off*_1_! = 0*xFF*)). Then, the second mutated file is marked as redundant because it is satisfied by ((*off*_1_! = 0*xFF*)). Since the third test case violates all the covered paths before, it is marked as significant, and the fuzzing engine performs a real execution within QEMU, and the new path (i.e., (*off*_1_ == 0*xFF*) ∩ (*off*_2_ == 0*xD*8)) is collected in the covered paths. After 7 mutations, our system successfully covers all paths in this sample code. Compared with traditional fuzz testing, we need only to perform 4 real executions. Since the time of the verification process of the constraint solver is shorter than a real execution in full system emulation QEMU, our approach can increase efficiency by reducing the number of executions. However, with the increasing number of covered paths, verifying all paths can bring more overhead. To avoid this, we collect a *hit count* for each path in the concrete execution. A path with a higher *hit count* means more test cases will be mapped to this path. Based on the *hit count*, we selectively verify only paths (i.e., hot states) whose *hit count* are above a predefined threshold.

### Selective branch query

As mentioned before, fuzz testing tends to get stuck when testing complex code regions. To avoid this, we leverage symbolic execution to assist fuzz testing to penetrate into deeper code spaces and improve the coverage.

**Fig 4:**
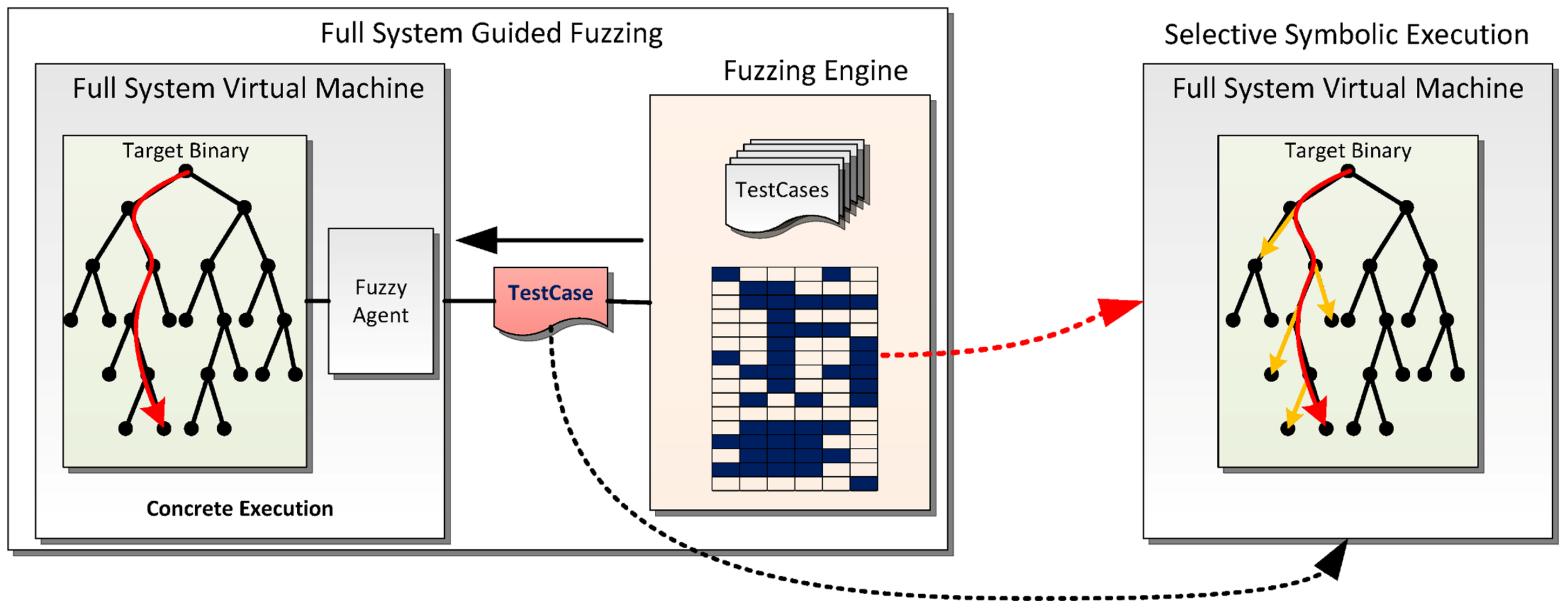
Framework of full system fuzz testing with S2E assisted. The red execution trace denotes the path of the current test case. And the yellow branches are the branches that the symbolic execution engine can cover.

We wish to utilize a symbolic execution engine that does not raise false alerts, thus avoiding generating useless test cases. Driller [16] has attempted to integrate concolic execution’s ability into fuzz testing, leveraging Angr [23] as its concolic execution engine. Angr is a user space level symbolic execution; it has to set all the environment variables as *unconstrained*, which may lose some critical paths when testing large-scale real-world software. S2E handles all cases correctly because it can emulate the full system environment. So, we choose S2E as our symbolic execution engine and leverage its highly accurate selective symbolic execution ability to assist the fuzzing engine and thus improve its coverage when testing complex code. Fig 4 illustrates our design, which integrates selective symbolic execution into fuzz testing.

Most of the time, we perform full system guided fuzz testing. As shown in Fig 4, when the fuzzing engine cannot produce any new coverage in a given budget time, the corresponding test case as well as the bitmap is sent to the selective symbolic execution engine to improve coverage.

The selective symbolic execution engine starts concolic execution with the template file (the red test case shown in Fig 4). The symbolic execution in our design is *scale controllable* as follows. For each symbolic branch, the engine checks whether the branch has already been covered; if not, the constraint solver is invoked to generate a new test case of this branch (marked as the yellow lines in Fig 4). Otherwise, symbolic forking is disabled on this branch to avoid overhead. After all the new branches are covered by the symbolic execution engine along with this template file, the new generated test cases are added to the test case queue of the fuzzing engine since each of them represents a new path.

Different loop times of a specific loop can influence program behavior in different ways. However, since the loop structure covers the same basic block transition edges, it may not be recognized as new coverage. Thus, loops should be carefully handled to represent the divergence of different loop times. AFL utilizes a “loop bucket” to reflect such divergence; it dissolves loop times into 8 buckets, i.e., [1, 2, 3, 4-7, 8-15, 16-31, 32-127, 128+]. Changes in the same bucket are not regarded as new behavior. Changes between different buckets are treated as new paths, and the test cases that trigger these changes are added to the test case queue. Loops also must be handled very carefully in symbolic execution, especially when the cycle control-variable contains symbolic data, which may trigger a path explosion. In our design for handling loops in symbolic execution, we first recognize all the loops in the target program by static analysis. The loops are then configured to help the symbolic execution engine. Algorithm 1 shows our loop handle method in symbolic execution engine.

**Algorithm 1:** Symbolic Loop Bucket

**Input:** Configured Loops *L*, Current Edge *CE* and Bitmap *B*_*p*_

**Output:** Generated test cases *t*_*slb*_

**1** **if *not*** *IsaLoopCycleEdge*(*L*, *CE*) ***or not*** *IsaSymLoop*(*CE*) **then**

**2**  return;

**3 end**

**4** *loopTimes* = 1;

**5** *UBs* = ParseUncoveredBuckets(*B*_*p*_);

**6 while** *TRUE* **do**

**7** **foreach** *ub in UBs* **do**

**8**  **if** *loopTimes* ***within*** *ub* **then**

**9**   *t*_*slb*_.add(GenerateTestcase());

**10**   *UBs*.remove(*ub*);

**11**  **end**

**12** **end**

**13** **if** *UBs* ***is*** *null* **then**

**14**  return *t*_*lsb*_;

**15** **else**

**16**  ExecuteOneCycle();

**17**  *loopTimes*+=1;

**18** **end**

**19 end**

According to the configured loops, we first determine whether the current edge (i.e., current branch) is a loop cycle edge and whether the loop is controlled by symbolic data (line 1-3). After confirming the execution flow is in a *sym-loop*, the state fork is disabled in already covered loop buckets, and once an uncovered bucket is reached, we generate a new test case and add it to the test case queue; this uncovered bucket is then removed from the uncovered loop buckets to avoid generating multiple test cases (line 7-12). After all buckets are covered, we break from the current loop to execute further code.

## Implementation

Ffuzz is built on top of the state-of-the-art genetic fuzzing framework AFL [3]. AFL uses user mode QEMU emulation to perform user space fuzz testing. In Ffuzz, we replaced the user mode QEMU by S2E. S2E is a dynamic binary analysis platform that utilizes selective symbolic execution to analyze whole software stacks at runtime. S2E is available for many instruction set architectures, such as X86 and ARM. The S2E reuses parts of the QEMU virtual machine, the KLEE symbolic execution engine [24] and the LLVM tool chain [25].

The fuzzing logic of AFL was not changed from an obvious way; we only added some extra code to communicate with S2E. Most of the work was done on the S2E side, where we added three main analysis plug-ins, i.e., *FuzzySearcher*, *ExceptionMonitor* and *PathRecorder*, as well as the *fuzzing agent*.

The *FuzzySearcher* is implemented to communicate with AFL and schedule all the execution states for S2E. It contains the implementation of all three mentioned optimizations. The *ExceptionMonitor* is used for monitoring all the exceptions. It parses the System.map file at the initialization phase so that we can intercept the process exit function and oops entrance function to monitor both the user and kernel space’s exception. The *PathRecorder* instruments the translation procedure of QEMU and splits our target process from the OS level to generate the bitmap, which is then transmitted to AFL to analyze whether the current test case is generated by reluctant mutation. The *fuzzing agent* is integrated as a component of QEMU to help the *FuzzySearcher* accomplish the task of executing the state schedule. These three plugins as well as the *fuzzing agent* consist of an additional 3100 lines of C/C++ code.

**Fig 5:**
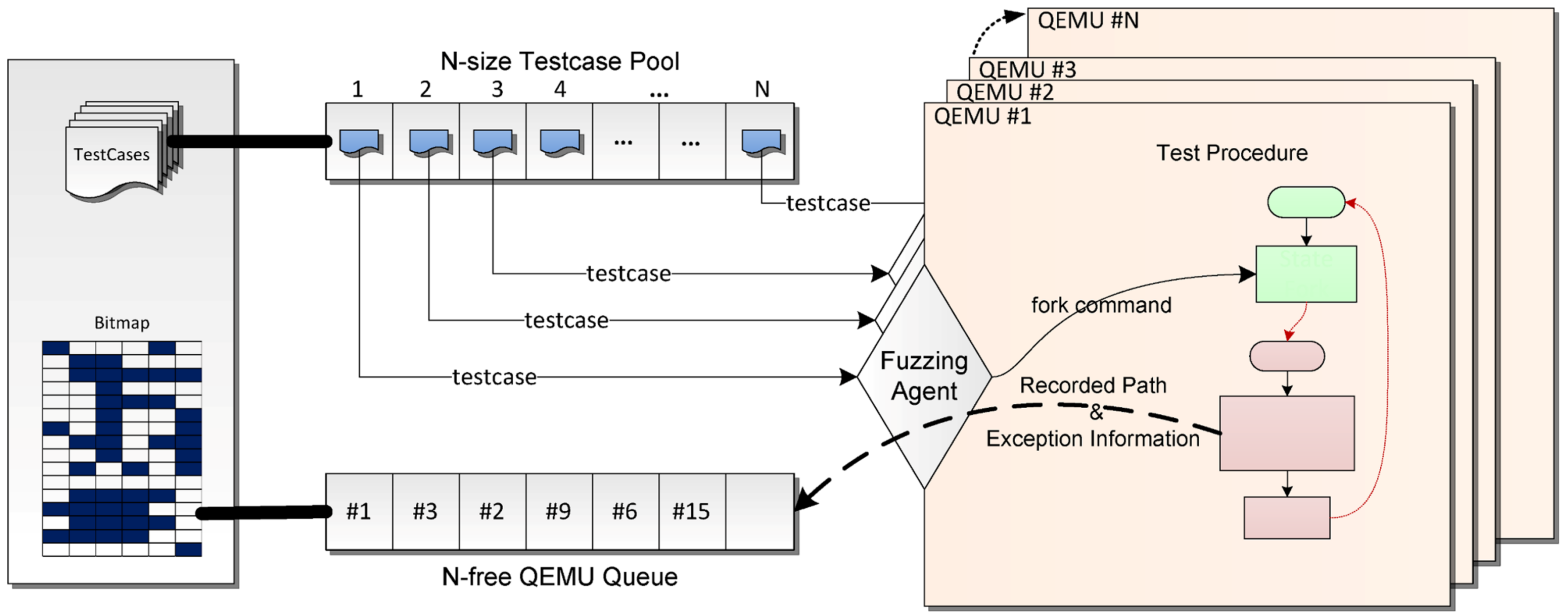
Pipeline fuzzing framework.

Although we have implemented the “speedup” optimizations as mentioned before, fuzzing the whole system is still much slower when compared with fuzzing in the user space. To improve efficiency, we extended the previous framework into a pipeline framework and also introduced computation cluster technology. Fig 5 shows our pipeline fuzzing framework.

After comparing the time consumption of mutation and testing, we realized that mutation is much faster than execution in full system mode QEMU. So, the fuzzing engine always waits for the QEMU instances to finish their testing jobs. Based on this acknowledge, we created a *N*-size test case pool for all the QEMU instances so that each one can fetch a test case independently to achieve a testing pipeline framework. We also shared the bitmap in the fuzzing engine between QEMU instances and maintained a *N*-free QEMU queue so that the fuzzing engine can determine which QEMU is free and ready to perform a new test.

Algorithm 2 shows the Pipeline control method in Ffuzz.

**Algorithm 2:** Pipeline control method

**Input:** Program *P*, Interesting test case queue *ITCqueue*

**1** **while** *TRUE* **do**

**2** *t* = FetchFromQueue(*ITCqueue*);

**3** $\bar{t}=\text{Mutate}(t);$

**4** *Q*_*i*_ = ReadQEMUQueue();

**5** **if** *HasNewCoverage*(*Q*_*i*_) **then**

**6**  AddToQueue(*ITCqueue*, *Q*_*i*_.*testcase*);

**7**  UpdateBitmap(*Q*_*i*_);

**8** **end**

**9** WriteToQemuPool($\bar{t}$);

**10** NotifyQemu(*Q*_*i*_);

**11 end**

We regard all the test cases that cover new paths as interesting test cases and collect them into a queue (i.e., *ITCqueue*). Each test case of the queue is fetched and mutated (Line 2&3). Then, the fuzzing engine reads the *N*-free QEMU queue to get a free QEMU instance (line 4); this operation is blocked if no QEMU instances are queued. Once a free QEMU instance (*Q*_*i*_) is found, the fuzzing engine collects the result of *Q*_*i*_ for the last test. If the last test case of *Q*_*i*_ triggers new program behavior, it is added to *ITCqueue*, and the bitmap of the fuzzing engine is updated to reflect this new behavior (line 5-7). After that, the newly generated test case (i.e., $\bar{t}$) is written to the work directory of *Q*_*i*_ to start a new testing (line 9&10).

## Performance evaluation

As far as we know, Ffuzz is the first platform to support full system software stack fuzz testing. We benchmarked Ffuzz on real-world binary software to evaluate its performance.

### Path discovery

We set up two groups of experiments to evaluate the path discovery ability of Ffuzz. We selected a JPEG-parsing program (rdjpgcom, version: 9, size: 29.4KB) to evaluate the testing speed, which is a key factor of fuzz testing. Then, we selected several examples of real-world binary software to evaluate the capability of path discovery.

All of the experiments were conducted on a machine with 16 cores and 32 GB memory, and the OS is Ubuntu 14.04 LTS running with Linux kernel 3.13.0.

Table 2 lists the execution speed of Ffuzz and vanilla AFL. From this table, we can see that Ffuzz reaches a half average execution speed of AFL, which works in user mode. Table 3 shows the number of basic blocks and instructions handled in full system and user mode, respectively. From the table, we can determine that the amount of instructions that Ffuzz handles is 354x more than user mode, which only needs to handle the instructions in the current running task. So, the testing speed of Ffuzz is **reasonable** when regarding the difference between full system and user mode.

**Table 2:** Execution speed* of FFuzz and vanilla AFL.

Tool	Speed	Mode
Ffuzz	331	full system mode
AFL	660	user mode

*Execution speed denotes the number of test cases executed per second.

**Table 3:** Basic blocks and instructions handled by full system and user mode.

Mode	Basic Blocks (#)	Instructions (#)
full system	789980	4739887
user mode	2231	13381

**Fig 6:**
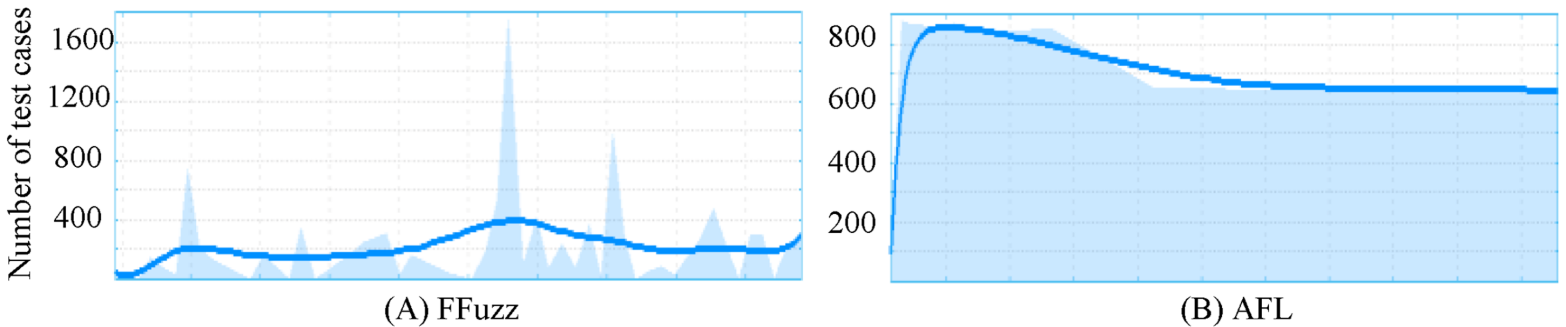
Details of execution speed for FFuzz and AFL in one hour. Figures are generated by afl-plot from AFL.

The details of the execution speed of Ffuzz and AFL are shown in Fig 6. From Fig 6(A), the speed of Ffuzz can reach a highest speed of 1750 test cases per second. Most redundant test cases are quickly filtered by the symbolic execution engine, which corresponds to the peak points. Remaining test cases are inspected under full system QEMU. The bottom of Fig 6(A) reflects such tests, which also shows that performing a test under full system QEMU is slower than in user mode.

**Fig 7:**
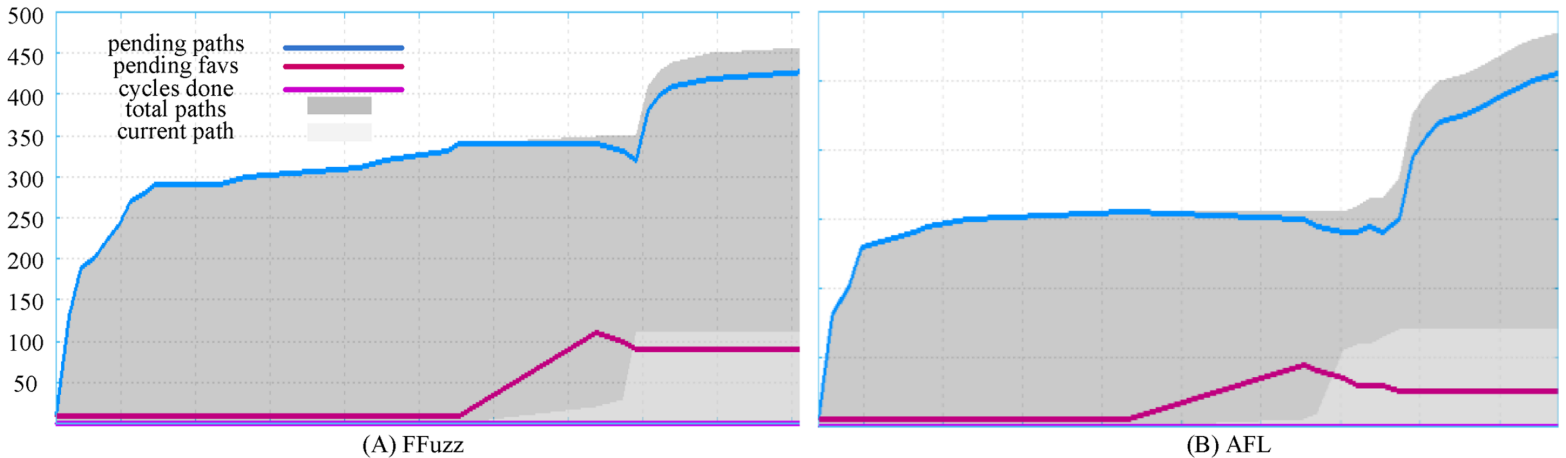
Paths* covered by FFuzz and AFL in one hours. *Path refers to the *unique path* from AFL. Figures are generated by afl-plot from AFL.

To demonstrate the ability of the path discovery of Ffuzz, we investigated the number of *unique paths* discovered by Ffuzz and vanilla AFL. Fig 7 shows the results of one hour’s test. From this figure, we can see that even though we are performing fuzz testing in full system mode, we can achieve the same tendency of unique paths as AFL in user mode.

We also benchmarked Ffuzz on several target programs (cat, rdjpgcom, gif2png, djpeg, wrjpgcom) to demonstrate the abilities of *FPD*(which introduces the peak points of Fig 6(A)) and selective branch query. Table 4 lists the testing result. Column 1 lists the target programs; column 2 shows the size of the target program; column 3 and column 5 present the number of test cases that are generated with symbolic execution assistance (SA) and without symbolic execution assistance (PF); column 4 and column 6 present the test cases that are executed with and without symbolic execution assistance; column 7 lists the filter percentages both for SA and PF mode; and column 8 shows the discovered unique paths for these two modes.

**Table 4:** Detail comparison between two different testing modes.

Target Program	Size (KB)	GenSA	ExeSA	GenPF	ExePF	FP (%)		TP (#)		Gain
						SA	PF	SA	PF	
cat	52	986048	253175	251697	251697	74.26	NULL	432	171	2.53
rdjpgcom	20.5	1127110	203342	206405	206405	81.96	NULL	475	152	3.13
gif2png	27	1268419	229092	235008	235008	81.94	NULL	542	201	2.70
djpeg	30	896535	186275	209097	209097	79.22	NULL	292	115	2.54
wrjpgcom	21	1370578	260078	260906	260906	81.02	NULL	413	167	2.47

Gen* denotes the number of test cases **generated** by *Symbolic Assisted*(SA) or *Pure Fuzzing*(PF); Exe* denotes the number of test cases **executed** by *Symbolic Assisted*(SA) or *Pure Fuzzing*(PF); FP refers to *Filter Percentage* and TP refers to *Touched Paths*. All the data are collected in one hour’s testing.

From the table, we can see that with one hour of testing, SA mode can touch more paths than PF mode, although the number of test cases that is executed for these two modes are at the same level (as shown in column 4 and 6); SA mode has discarded redundant test cases and only kept the ones that have a greater probability of triggering new paths. The data onto column 7 show that, under SA mode, an average of 80% of generated test cases will not actually be executed to avoid testing overhead. We can also derive from column 9 that with the help of symbolic execution, Ffuzz can discover 2.74x more paths than without symbolic execution.

### Bug discovery

We have investigated the bug discovery ability of Ffuzz on both Ring 3 and Ring 0 programs.

The user space target programs are selected in Juliet test suite v1.2 for C&CPP [26], which can be classified by different vulnerability types. Each program of our evaluation target has a good function and a bad function. The good function demonstrates the normal behavior, while the bad function represents a specific bug or vulnerability. In our effectiveness evaluation, we run each program with both a good function and bad function invoked to demonstrate the false positive and false negative.

As mentioned previously, the first optimization of Ffuzz deals with a deterministic program, so we filtered the test suite and dropped off all the programs that contain random-like functions or instructions. Among all the deterministic programs, some do not receive input from outside, which means their behavior is fixed. We discarded the fixed behavior program since they cannot be affected by input data.

Table 5 lists the selected programs and the evaluation results of Ffuzz. From this table we can see that Ffuzz detected all vulnerabilities without raising any false positives or false negative.

**Table 5:** Targets filtered from Juliet benchmark.

Bug Type	Total Vuls(#)	Detected(#)	FP	FN
CWE121_Stack_Based_Buffer_Overflow	172	172	0	0
CWE122_Heap_Based_Buffer_Overflow	172	172	0	0
CWE369_Divide_by_Zero	172	172	0	0
CWE680_Integer_Overflow_to_Buffer_Overflow	328	328	0	0

For kernel space testing, we selected the e1000 driver in the Linux kernel to observe whether Ffuzz can detect the vulnerability referred to as CVE-2009-1385 [27]. CVE-2009-1385 results from an integer underflow in the e1000_clean_rx_irq function of drivers/net/e1000/e1000_main.c in the e1000 driver in the Linux kernel before 2.6.30-rc8, the e1000e driver in the Linux kernel, and Intel Wired Ethernet (aka e1000) before 7.5.5, which allows remote attackers to cause a denial of service (kernel panic) via a crafted frame size.

Table 6 shows the result of real-world device driver testing; we spent 7.8 hours (executed 5278549 test cases) to find this bug with Ffuzz. The result demonstrates that Ffuzz is capable of detecting the vulnerabilities in the kernel space.

**Table 6:** Result of real-world device driver testing.

Driver	Version	Vulnerability	LoC	Time	Test Cases	Detected
e1000	7.5.5	CVE-2009-1385	13,971	7.8 hours	5278549	Y

## Discussion

### Comparison with other tools

Fuzzing in full system mode is still challenging as we need to leverage a full system emulator to monitor exceptions, in particular in coverage-based fuzz testing. Table 7 shows some of the results of a basic comparison between state-of-the-art fuzz testing tools, including AFL and Driller, TriforceAFL [28], and Ffuzz Ffuzz.

**Table 7:** Comparison with AFL, Driller and TriforceAFL.

Tool	SA	SC	FS	TEC
AFL	N	NULL	N	Y
Driller	Y	N	N	Y
TriforceAFL	N	NULL	Y	N
Ffuzz	Y	Y	Y	Y

SA denotes whether the tool is *Symbolic Assisted*; SC represents whether the tool maintains *Symbolic Consistency*; FS denotes whether the tool supports *Full System* testing; and TEC denotes whether the tool maintains *Testing Environment Consistency*.

We can see from the table that both Ffuzz and Driller are concolic execution assisted. Since Driller is working in user mode, it cannot ensure the consistency of symbolic execution as the constraint information is lost when the execution enters into the kernel space. Unlike Ffuzz, TriforceAFL does not ensure each test case will be investigated in the same environment (i.e., *TEC*). This creates problems when the testing program is implemented as a state machine (e.g., network protocol) because the results of earlier test cases may affect later ones. Ffuzz eliminates this problem by forking a new state for each test case.

Meanwhile, although Ffuzz now focuses on *nix system testing, since Ffuzz is built on top of a full system QEMU, it would not be difficult to test other platforms like Windows and Android. It would only be necessary to modify the Full System Exception Monitor module to make it adaptable to a specific platform.

### Future work

Machine learning especially deep learning has gained more attentions in both research and industrial communities and it has achieved many attractive results in many different areas [29–31]. And such method is also investigated to see how to apply it on software testing and vulnerability detection [32–34]. Based on such work, we propose that in the future, machine learning could be ported to binary software vulnerability detection by cooperating with guided testing techniques. Since machine learning can raise many false positives, one can leverage guided dynamic symbolic execution to mitigate the false positives and verify the existence of potential vulnerabilities.

## Conclusion

In this paper, we put forward a novel method to detect bugs in full software stacks, including the user application, device driver and OS kernel. Our method builds on top of fuzz testing and selective symbolic execution. We perform full system dynamic binary instrumentation in our testing and monitor the exception signals in full system scale to capture bugs. Meanwhile, we have proposed optimizations to improve the performance of full system testing, including Fast Path Discovery, Selective Symbolic Branch Query. Fast Path Discovery collects the constraints of paths that are already discovered and utilizes the constraint solver to filter the reluctant mutations by constraint verification. Selective Symbolic Branch Query tries to discover more paths when fuzz testing gets stuck. Every time that fuzz testing cannot find more paths, we leverage selective symbolic execution to generate test cases that could touch the uncovered paths.

We implemented Ffuzz Ffuzz and demonstrated the effectiveness and efficiency through several experiments. The results show that Ffuzz can discover software bugs that exist in binary executables in a full system software stack effectively and efficiently.
